# The Geological Observer

**Published:** 1852-02

**Authors:** 


### REVIEWS.

Art. IV.—*The Geological Observer.* By Sir Henry T. De La Beche, C. B., F. R. S., &c. Director General of the Geological Survey of the United Kingdom. Philadelphia: Blanchard & Lea. 1851.

The progress made by Geological science since its great elemental truths were first announced by Dr. Hutton, before the Royal Society of Edinburgh, in 1785, is one of the most surprising evidences of intellectual activity, energy and power that we remember in scientific records. The profound and unanswerable truths first promulged by Dr. Hutton, though fully and ably sustained by Professor Playfair, did not save the infant science from, what was supposed to be, a religious warefare. It assumed the worst possible phase that the spirit of the times would permit. It gloried in darkness, and blazed with denunciation, dark, bitter, unrelenting and devilish. In the full light of geological truth that now streams in uninterrupted fullness from every quarter of civilization, it is difficult to realise the fierceness and the folly that opposed the calm, philosophical and truthful views contained in Dr. Hutton's celebrated "Dissertation on the Theory of the Earth." For awhile, according to Professor Lyell, it was better for a man to have a stain upon his moral reputation than to yield assent to the geognostic views of Dr. Hutton. The clergy leveled broadsides of scripture at the rash adventurers, the church took the alarm, and denounced anathemas against the new irruption of Gibbons, Voltairs, Paines, and Rousseaus who were said to have assailed revelation in the guise of philosophy.

Despite, however, of the argumentation we have rather hinted at than described, the new philosophy made rapid progress. The earth teemed with its truths, and the ardent and admiring disciples of Dr. Hutton, and a nobler band never gathered under a scientific banner, were enabled to pile proof upon proof, to such an extent as to sweep all opposition into annihilation. But we know of no stronger evidence of the nature of the impenetrable veil of darkness which the mists of religious prejudice can make, than is furnished in Dr. Buckland's *Reliquæ Diluvianæ.* In that work Dr. Buckland was determined to make the truths of geology fit his notions of the Deluge, and he permitted himself to indulge in the most foolish diatribes against the labors of Cuvier, which he must have remembered with shame and confusion while he was writing, subsequently, his masterly Bridgewater treatise. In that he wisely forgot his Deluge notions, and permitted geology to speak its truths, without attempting to mix them up with matters that had no kind of connection with them.

The vastness and all-absorbing interests connected with geology commend that science to the study of every human being that has any fondness for science of any kind. Its profuse and varied riches enable us to trace creation almost from its very dawn, through all its various stages, up to the time when history undertook its work with the earth. In surveying the crypts of the earth, the mind expands and elevates itself to a relationship with the grandeur and magnificence of the theme before it; from the gushing streams of knowledge that pour forth in every direction a perpetual joy is furnished; and from the whole survey, man feels that he knows his Creator better from a study of the revealments of fossils and strata of rock, than he could know him from written revelation alone. To the medical inquirer these studies are of the profoundest interest, and we envy not that medical mind that takes no pleasure in these things. Every truth in nature has its value for the medical philosopher, and he who is the most deeply imbued with the truths that belong to each department of nature, most perfectly fulfils the demands of philosophy.

It is not our purpose to write an introduction to the merits of Geology. We hope the day for a defence of or a plea for the merits of science has gone, and that those who acknowledge their ignorance of its truths have some more truthful excuse than is found in any depreciatory statements respecting it.

To those who feel any desire to enter upon the study of geology under the guidance of a most intelligent instructor; one who is not only capable of instructing, but of delighting at the same time, we warmly commend Sir Henry de la Beche. His name and his labors have long been familiar to the students of geology, and he has built up for himself, a reputation that will live as long as the science of geology shall attract the attention of men.

We purpose now, not to analyse the work before us, but to indicate some of the sources of pleasure, and some of the characteristics of useful and invigorating knowledge that may be confidently sought in this volume.

The author thus announces the aims and objects of his book:—"The following work was undertaken in the hope that the experience of many years might assist and perhaps abridge the labors of those who may be desirous of entering upon the study of geology, and especially in the field. Its object is, to afford a general view of the chief points of that science, such as existing observations would lead us to infer were established; to show how the correctness of such observations may be tested; and to sketch the directions in which they may apparently be extended."

We know of no other work that fulfils the objects presented in these remarks as perfectly as the book before us. He that would understand the geognostic condition of the globe, both past and present, should seek it in the ample pages of De la Beche's Geological Observer.

The Introduction to the scientific portions of the work is occupied with an examination of the nomenclature of Geology, and it is certainly desirable that a more perfect system than now obtains should be introduced in that department. A great deal of confusion arises from naming groups of rock from some special locality, by which a necessity is imposed for the study of the nomenclature of each section of the world. The Devonian, Jurassic, Cambrian and Silarian groups of fossils are thus named from special localities, instead of from certain characteristics that would suit those groups, no matter where they might be found.

The relations of the characteristics of soils to vegetable and animal life are of great value to the scientific physician, and as they are not elucidated well by any other science than geeology, they constitute a plea for a thorough understanding of all that is known on the subject. They will not enable a man to discriminate between the various forms of fever, to understand the phenomena of inflammatory actions, to amputate a limb, to open abscesses, or to lance gums, but these are but portions of the claims of medicine to public confidence. An understanding of the whole science of hygiene; the etiology of diseases, and of the essential laws of sanitary regulations, is an essential portion of the paraphernalia of the true physician, and this understanding is inseparably connected with geological studies. Pretenders to geological science, for it has its pretenders as every other useful pursuit has, have ridiculously attempted to make out the originating meanderings of epidemic disease, by certain geological formations, but these follies never were undertaken by the masters of the science, and their failure reflects discredit on the authors of the folly, not on the science itself. But if the production of any form of disease is the result of terrestrial influences, those influences are connected with the soil, and it forms a proper subject of medical study.

De La Beche very properly opens the science of geology, with the disintegration and decomposition of rocks, and the growth of soils as a consequence of such decomposition. And the medical reader is prepared to expect that etiological facts will be modified materially by the character of the rock, the decomposition of which makes a special soil. Those diseases that spring from the decay of vegetable matter will be more abundant in the region of soils formed by the decomposition of limestone rocks, than where the soils are made of the decompositions of sand-stones, or granitic rocks. The decomposition of rocks containing oxides of iron also shows modifications of special interest. These various decompositions are handled, geologically, with singular ability, in the work before us. It is the duty of the physician first to master these truths, and then he may hope to apply them to advantage.

The whole geognostic panorama passes in review before the student of De La Beche's sections. Thus we have, "the removal of parts of rocks by water, soluble substances, carbonate of lime, travertine, analysis of running water, hot springs, geysers, springs on the out crop of strata, springs on faults, cause of land-slips, transport of detritus by rivers, deposit of detritus in valleys, action of rivers on their beds, removal of lakes by river action, formation and discharge of lakes." The laws of drainage, thus displayed by nature, are of vast interest to him who wishes to be useful in the sanitary regulations of communities, and those laws are developed in the sections we have indicated, with great force, clearness and perspicuity.

But let us resume the subjects of the work before us, and follow our author through "lacustine deposits, action of the sea on coasts, difference of tidal and tideless seas, the unequal abrasion of coasts, shingle beaches, coast sand hills, contemporaneous deposits of mud, sand and gravel, influence of ice in the Baltic, gulf of Mexico and Mississippi. Bars at river mouths, deposits in estuaries, influence of waves, form of the sea bed round the British isles, influence of currents, distribution of sediment over the floor of the ocean, chemical deposits in sea, and calcareous deposits." These prepare us for the phenomena of "the preservation of remains of existing life in mineral matter, plants and vegetable matter, bogs, Dismal swamp, rafts in the Mississippi, animal remains on the land, vertebrata, insects, land molluscs, effects of showers of volcanic ashes, foot-prints in mud, marine deposits and remains, modifications of conditions on the coasts of America, of the Pacific ocean, of the Indian ocean, coasts of Africa and Europe, Arctic sea, distribution of marine life, modifications from temperature and pressure, from light and supply of air, Sir E. Forbes, on the Ægean sea, zones of depth, zones of depth in the British seas, organic remains deposited in the deep ocean, corals in all their forms and formations." This section contains matters of great interest to the physician. In one item,—the influence of light and air, there is a volume of instruction, even to the mere bed-side physician. It is not very complimentary to medical science, that Cuvier was first permitted to enunciate the universal law—"that all animals are vigorous in proportion to the amount of oxygen they consume," a law that cannot be too frequently before the mind of the physician in the treatment of disease.

Let us pursue the objects on which our author teaches how to observe geologically.

The 10th section is devoted to the following subjects: "transportal of mineral matter by ice, height of snow line, glaciers, cause of the movement of glaciers, grooving of rock by glaciers, Arctic glaciers reaching the sea, northern icebergs and their effects, and Arctic glaciers and ice barriers, transportal of detritus by river ice, geological effects of coast ice, effects of grounded icebergs, general geological effects of ice, influence of a general increase of cold, modifications of temperature from changes in the distribution of sea and land, effects of gradual rise of the sea bottom strewed with ice—transported detritus, increase of Alpine glaciers, transportal of erratic blocks by glaciers, elevation of boulders by coast ice during submergence of land, Arctic shells in British deposits, evidence of a colder climate in Britain, extinct Siberian elephant, changes of land and sea in Northern Europe, extinction of the great Northern mammals, range of the mammoth, frozen soil of Siberia.

Section 11th discourses in ample detail, "on ossiferous caves and osseous breccia, former connexion of Britain with the Continent, mammoth remains in the British seas, mud in ossiferous caves, human remains in Paviland cave, and in ossiferous caverns, pebbles in ossiferous caves, deposits in subterranean river channels, osseous breccia in fissures, changes in the entrance to caves, occurrence of mastodon remains, extinct mammals of France."

The 12th, 13th, and 14th sections are devoted to volcanic phenomena and earthquakes.

The 15th section discusses the following interesting subjects: "Quiet rise and subsidence of land, rise and depression of the coast in the bay of Naples, elevation and depression of land from increase and decrease of heat, gradual rise of land in Sweden and Norway, raised coasts in Scandinavia, gradual depression of land in Greenland, movements of coasts in the Mediterranean, unstable state of the earth's surface."

Section 16th is devoted to the following themes: "Sunk (submarine) forests and raised beaches, sunk forests of Western Europe, sunk forests beneath the sea in roadsteads, mode of occurrence of sunk forests, localities of sunk forests, remains of mammals and insects in sunk forests of Western England, foot-prints of deer and oxen amid the rooted trees of sunk forests in South Wales, raised beaches, detritus, &c.

The 17th section developes certain features of temperature that are not so familiarly known as they should be. The subject is divided into the following branches: "Temperature of the earth, temperature of different depths in Siberia, temperatures found in Artesian wells, heat of waters rising through faults and other fissures, variable temperature from unequal percolation of water through rocks, temperature of waters in limestone districts."

Section 18th contains groups of facts of the profoundest interest to the geological student. It embraces the following themes: "Mode of accumulation of detrital and fossiliferous rocks, detrital rocks, chiefly old sea bottoms, mixture of beds with and without fossils, variable mode of occurrence of organic remains among detrital and fossiliferous rocks, beaches on the shores of land at the time of the Siberian deposits and old red sandstones, beaches of the new red sand-stone series, beaches at the time of the lias, boring molluscs of the inferior oolite period, overlap of the inferior oolite, land of the time of the lias, evidence of land from fresh water deposits, effects produced on coasts, rivers, and lakes, by continued elevation of land above sea, elevation of land over a wide area, effects of closing the Straits of Gibralter, lakes on the outskirts of mountains, mixture of organic remains of different periods from submergence of land, variable effects of submergence of present dry land, evidence afforded by the coal measures, stigmaria beds in the coal measures, stems of plants in their positions of growth in the coal measures, filling up of hollow vertical stems, and mixture of prostrate plants with them, growth of terrestrial plants in successive phases in the coal measures, false bedding in coal measure sand-stones, surfaces of coal measure sand-stones, drifts of matted plants in the coal measures, extent of coal beds, partial removal of coal beds, channels eroded in coal beds, lapse of time during deposit of coal measures, pebbles of coal in coal accumulations, marine remains in part of the coal measures, gradual subsidence of delta lands, fossil trees and ancient soils, Isle of Portland, conditions under which the ancient soils and growth of plants were produced at Portland, raised sea-bottom round British islands, overlap of cretaceous beds in England, foot-prints of air-breathing animals on surfaces of rocks, rain marks on surfaces of rocks, elevation or depression of the bottom in the ocean, character of the surfaces of rocks, friction marks on rock surfaces, ridged and furrowed surfaces of rocks, effects of earthquakes on sea-bottoms, mode of occurrence of organic remains, distribution of organic remains, effect of the rise and fall of land on the distribution of organic remains, distribution of organic remains with respect to different kinds of sea-bottoms, infusorial remains, caution as to forms of life supposed characteristic of different geological times.

Sections 19th and 20th, treat of igneous products of earlier date than those of modern volcanoes, of the various phenomena of igneous rocks, elvans, dykes, granitic and porphyry dykes, composition of igneous rocks, serpentine and diallage rocks, olivine, syenite, hornblende, minerals entering into the composition of igneous rocks, central fractures, phosphate of lime in rocks, crystaline phenomena in rocks, mineral matter transmitted into altered rocks, mica slate and gneiss, cleavages in their varieties, complications of bedding, jointing and cleavage.

Sections 21st and 22d possess all the interest of the most absorbing novel, with all the substantial realities of science. They develop the following matters of interest: "Bending, contortion and fracture of bedded rocks, earth's radius compared with mountain heights, the volume of the earth compared with mountain ranges, effects of a gradually cooling globe, mountain ranges viewed on the large scale, directions of mountain chains, conditions effecting the obliteration or preservation of mountain chains, binding and folding of deposits in the Appalachian zone, North America, flexures and plications of rocks in the Alps, igneous rocks amid contorted beds, contorted coal measures of South Wales, range of mineral veins and common faults in South Western England, filling of fissures and other cavities with mineral matter, sulphurets of lead, copper, &c., replacing shells, filling of minor fissures, solubility and deposit of mineral matter in fissures, deposits from solutions in fissures, frequent occurrence of sulphur, arsenic, &c., with certain metals in mineral veins, character of metaliferous veins amid associated dissimilar rocks, condition of mineral veins traversing elvan dykes, influence of different rocks traversed on the mineral contents of fissures, mode of occurrence of lead ores amid the lime-stones and igneous rocks of Derbyshire, 'flats' of lead ore in lime stone districts, metaliferous deposits in the joints of rocks," and so on through all the geological phenomena of metals.

Section 23d is devoted to the following subjects: "Partial removal of rocks, great denudation arising from the action of breakers, ancient exposure of the coasts of the area of the British islands to the Atlantic, island masses of deposits left by denudation, new slices of land now being cut away by breaker action, amount of matter removed by denudation, needful attentions to the greater geological problems."

We have thus endeavored to direct the attention of inquirers into geological science, to the ample resources of the work before us. It would be difficult to point to a book of profounder interest, and the geological student may resort to its pages under the confident assurance that he will find abundant knowledge conveyed in the most pleasing and impressive manner. Medical men should awaken to the importance of this kind of knowledge. It is shameful that thousands of them are passing their lives in the midst of the greatest treasures, which they are no more capable of understanding than the dialects of the Arian language. Kentucky, for instance, is one of the finest fields in the world for geological science, but what is Kentucky science doing for this noble field? Almost nothing.

Scientific men of every locality owe a debt not only to science, but to their scientific brethren, scattered over the world, and for this reason, we think that Kentucky should not much longer remain a sealed book to the world, nor should the scientific men of the State, by their apathy and neglect, permit aliens to come in and open these fountains of knowledge.

A Natural History Society has been recently commenced in Louisville, Ky., under very favorable auspices. The museum, which has already begun to grow, will be extensive, rich in various material, and admirably arranged. We earnestly hope that, at least, every Kentucky reader of these remarks will consider himself a special committee to contribute to the growth and value of this museum. The fossils, minerals, fauna and flora of Kentucky should be investigated and made known. We trust that these hints will neither be forgotten nor neglected.

We again and again commend De La Beche's great work to the attention of our readers. If each medical man in Kentucky could be induced to read that book, we should have no kind of fear for Kentucky geology. In addition to the perspicuous language of the author, each subject is elucidated by an excellent series of illustrations. The work may be obtained of Beckwith & Morton.

